# Supplementary material for: Constitutively bound CTCF sites maintain 3D chromatin architecture and long-range epigenetically regulated domains
Source: Nat Commun. 2020 Jan 7;11:54. doi: 10.1038/s41467-019-13753-7 (PMC6946690; doi:10.1038/s41467-019-13753-7)
Supplement: Supplementary file 2 — Supplementary Information [file 41467_2019_13753_MOESM2_ESM.pdf]

# Constitutively bound CTCF sites maintain 3D chromatin architecture and long-range epigenetically regulated domains

Khoury et al.

**Supplementary Information**

# Supplementary Figure 1

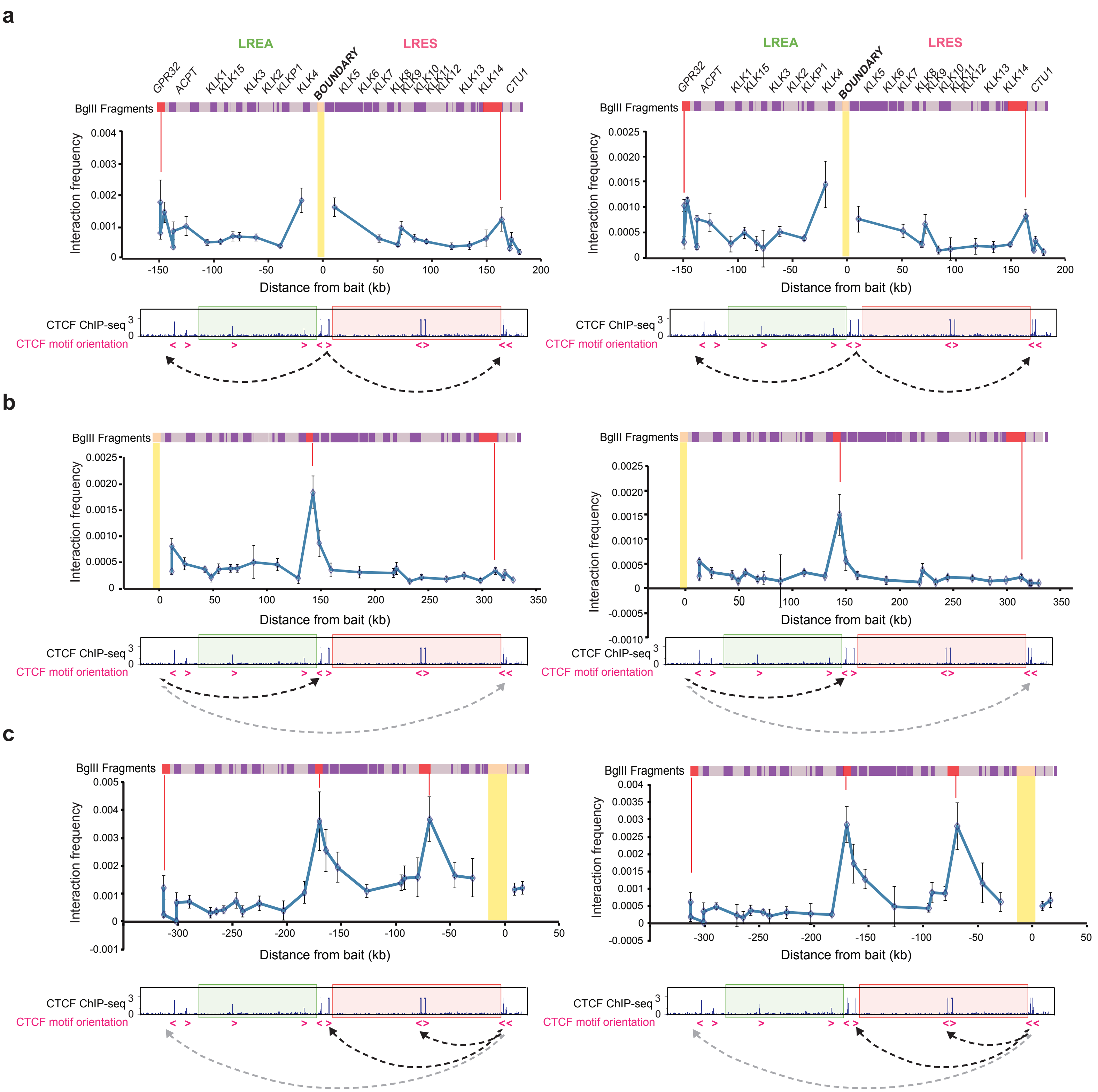


**Supplementary Figure 1: Biological replicates of 3C-qPCR at KLK locus.**

**a**. 3C-qPCR at the KLK locus. Bars above the graph illustrate BglII fragments across the region. The 3C bait fragment is indicated by the yellow bar and interacting fragments are shown by red lines (149.2kb upstream from bait, 163.8kb downstream from bait). The dotted line arrows demonstrate the loops indicated by the interaction data. Error bars represent SE.

**b**. As in Supp. Figure 1a, utilising upstream interacting fragment from Figure2a as bait. Interacting fragments are 143.2kb and 313.0kb downstream from the bait.

**c**. As in Supp. Figure 1a, utilising downstream interacting fragment from Figure2a as bait. Interacting fragments are 68.9kb, 169.8kb and 313.0kb upstream from the bait.

# Supplementary Figure 2

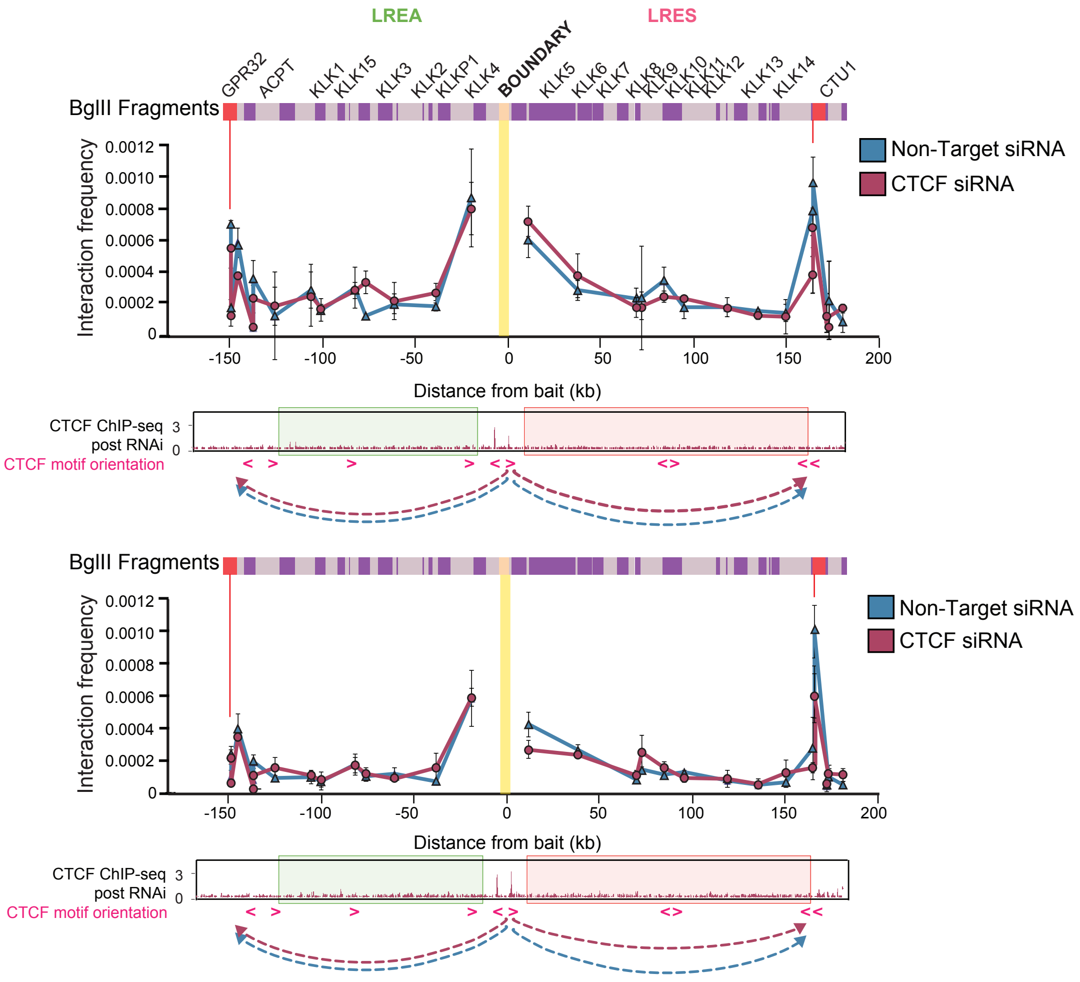


**Supplementary Figure 2: 3C-qPCR at KLK locus following 144hrs of CTCF siRNA.**
Biological replicates of 3C-qPCR at the KLK locus, in LNCaP cells following 144hrs of CTCF RNAi. Bars above the graph illustrate BglII fragments across the region. The 3C bait fragment is indicated by the yellow bar and interacting fragments are shown by red lines. Error bars represent SE. The decreases in looping interaction frequency between control and CRISPR conditions are represented schematically with dotted blue and pink line arrows, respectively.

# Supplementary Figure 3

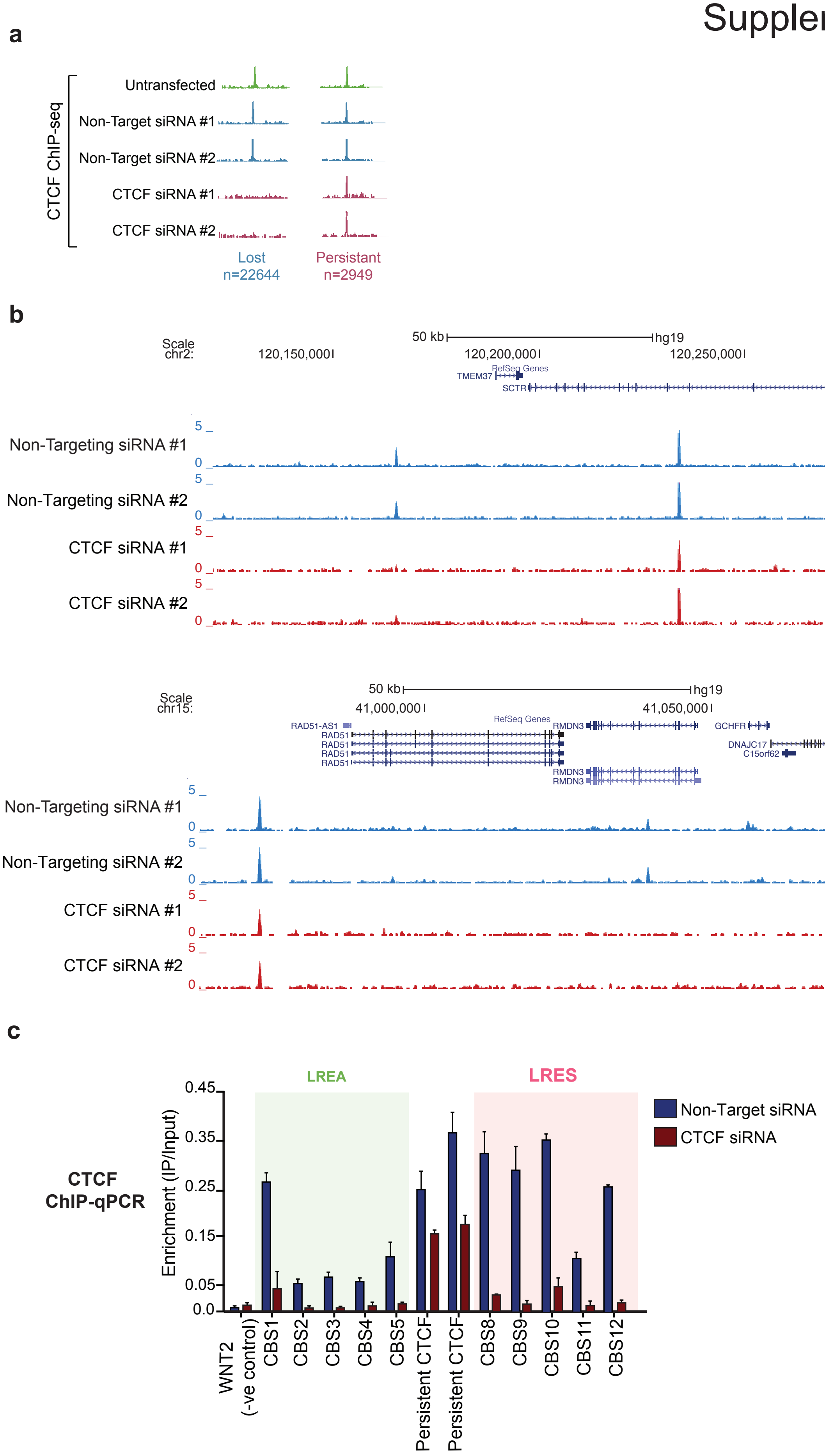


**Supplementary Figure 3: Subsets of CTCF binding sites following CTCF RNAi depletion.**

**a**. Schematic definition and quantification of 'lost' and 'persistent' CTCF sites.

**b**. UCSC genome browser screenshots demonstrating maintenance and loss of CTCF binding following 144hrs of CTCF RNAi.

**c**. CTCF ChIP-qPCR assaying all CTCF binding sites at KLK locus performed after 144hrs of CTCF RNAi in LNCaP cells.

# Supplementary Figure 4

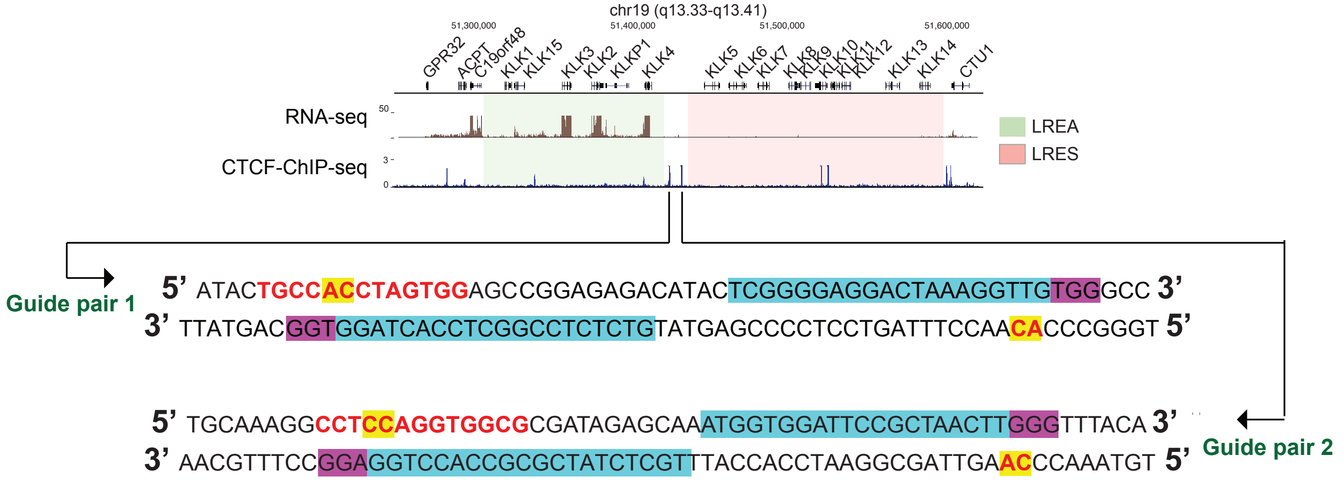


**Supplementary Figure 4: CRISPR-Cas9n assay design.**
CTCF motifs corresponding to persistent CTCF sites are in red lettering. sgRNAs designed to target the forward and reverse strands are highlighted in blue and adjacent PAM sequences in purple. Red text with yellow highlight indicates Cas9n cut sites.

# Supplementary Figure 5

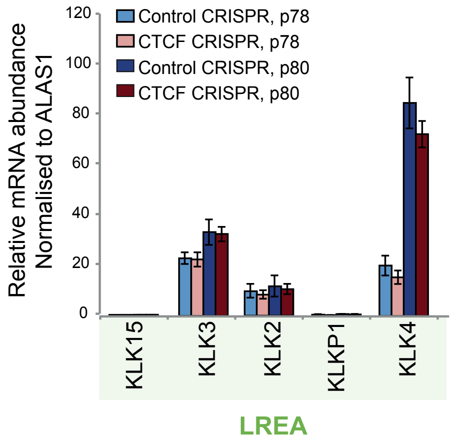


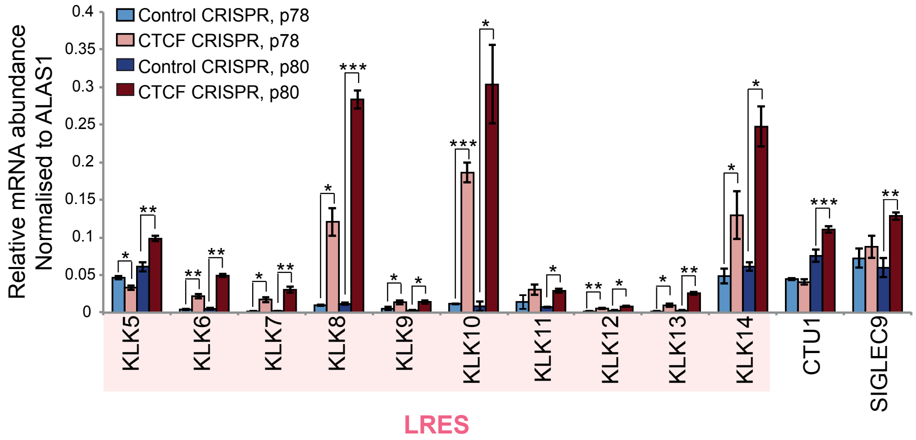


**Supplementary Figure 5: Quantification of gene expression following CRISPR-Cas9n of persistent sites at KLK locus**.

qPCR assaying mRNA levels of all genes within the KLK region on samples harvested post CRISPR-Cas9n addition. LREA genes remain unchanged, while LRES genes are all up regulated at both time points assayed. An ANOVA, followed by Tukey post hoc comparison was used to assess significant differences between Control CRISPR (n=3) and CTCF CRISPR (n=3) gene expression at each time point. * denotes p-value <0.05, ** denotes p-value <0.005, *** denotes p-value <0.0005. Source data are provided as a Source Data file.

# Supplementary Figure 6

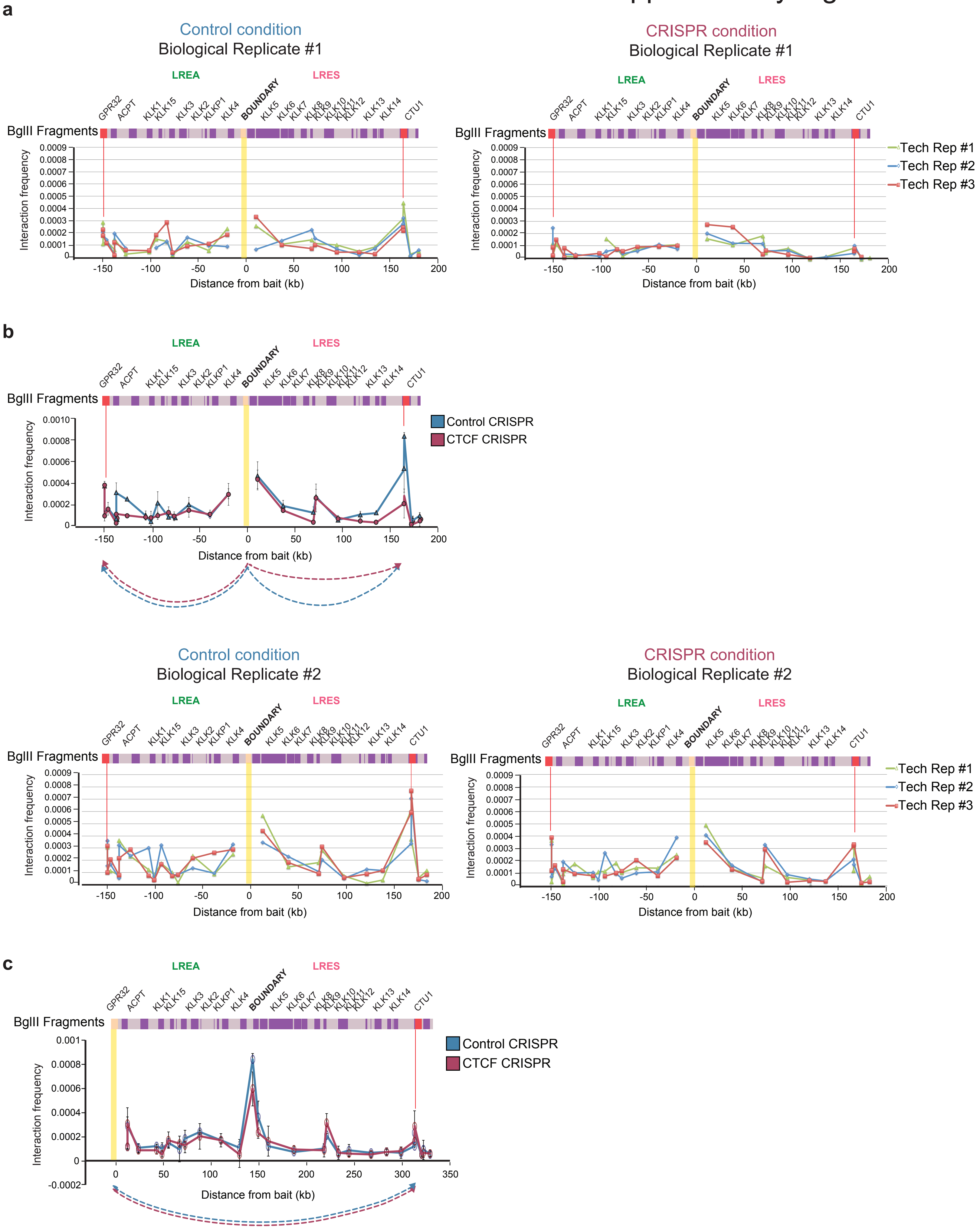

**Supplementary Figure 6: Chromatin conformation following CRISPR-Cas9n of CTCF persistent sites**

**a**. Individual technical triplicates (x3) of 3C-qPCR at the KLK locus for biological replicate #1 summarised in Figure 5d. Left panel: Control condition. Right panel: CRISPR condition.

**b**. Top: Biological replicate #2 of 3C-qPCR at the KLK locus following CRISPR of persistent sites. Bars above the graph illustrate BglII fragments across the region. The 3C bait fragment is indicated by the yellow bar and interacting fragments are shown by red lines. Error bars represent SE between technical replicates (x3). The decreases in looping interaction frequency between control and CRISPR conditions are represented schematically with dotted blue and pink line arrows, respectively.

Bottom: Individual technical triplicates (x3) of 3C-qPCR at the KLK locus for biological replicate #2 summarised above. Left panel: Control condition. Right panel: CRISPR condition.

**c**. Reciprocal 3C qPCR utilising upstream interacting fragment identified in Supp. Fig. 6a as bait. Bars above the graph illustrate BglII fragments across the region. The 3C bait fragment is indicated by the yellow bar and the interacting fragment of interest (313.3kb downstream) is shown by a red line. The dotted arrows schematically demonstrate no change to the level of interaction between bait and 313.3kb downstream. Error bars represent SE.

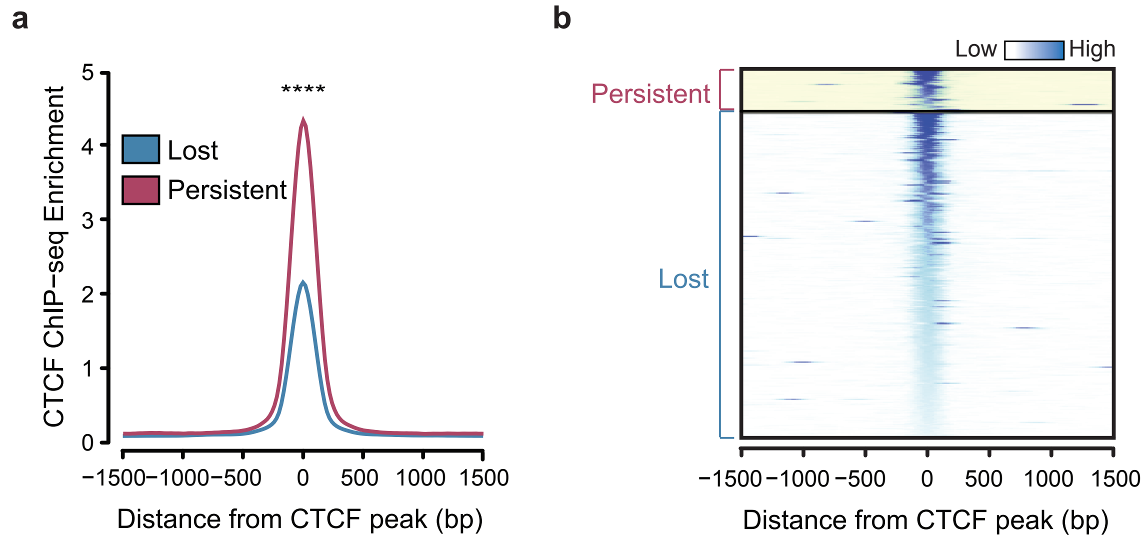


**Supplementary Figure 7: Binding strength of persistent and lost CTCF sites**

**a**. Wild-type LNCaP CTCF sites were subset into two groups according to whether peaks were persistent (purple) or lost (blue) following CTCF RNAi. Average ChIP-seq binding intensities for lost and persistent sites were plotted according to distance from CTCF site. Persistent sites had an average binding intensity 2-fold greater than lost sites (two-tailed t-test, ****$p < 0.0001$).

**b**. Heatmap showing intensity of CTCF binding for all CTCF sites in lost (no shading) and persistent (yellow shading) subsets defined in Supp. Figure 7a.

Supplementary Figure 8

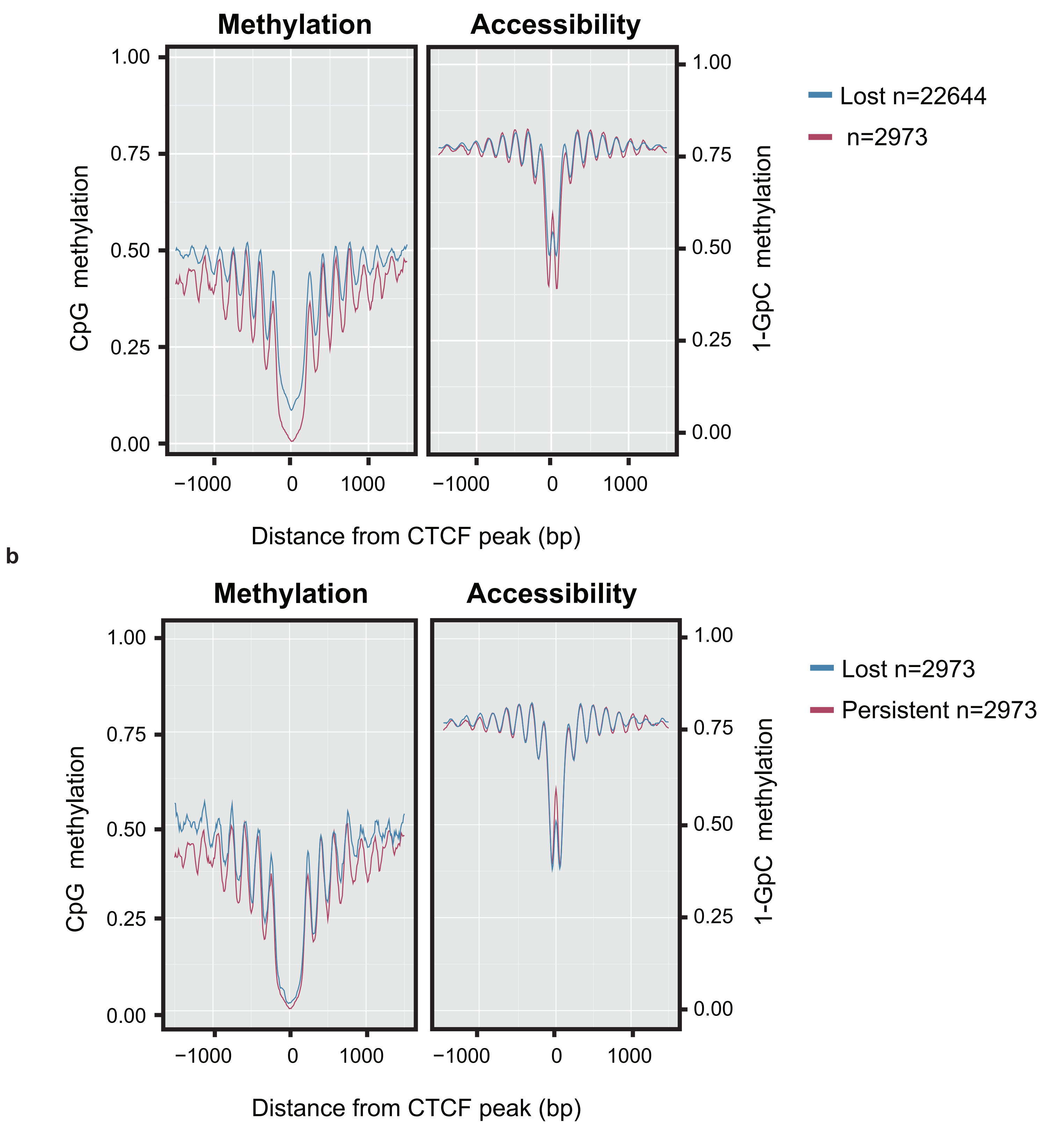


**Supplementary Figure 8: Methylation and accessibility of persistent and lost CTCF sites**

**a**. Wild-type LNCaP NOMe-seq data was subset into two groups according to whether peaks were persistent (purple) or lost (blue) following CTCF RNAi. (Left panel). NOMe-seq demonstrates that CpG methylation is more depleted at persistent CTCF sites compared to lost sites. (Right panel). Plotting 1-GpC methylation revealed that the DNA immediately overlapping the CTCF sites is more accessible at persistent sites than lost sites.

**b**. NOMe-seq data for lost CTCF sites was further down-sampled to the 2973 CTCF sites showing the strongest binding intensity. (Left panel). NOMe-seq demonstrates that CpG methylation is similar between the persistent sites and down-sampled lost subset. (Right panel). Plotting 1-GpC methylation revealed that the DNA immediately overlapping the CTCF sites is more constantly bound in the persistent subset when compared to the lost subset.

Supplementary Figure 9

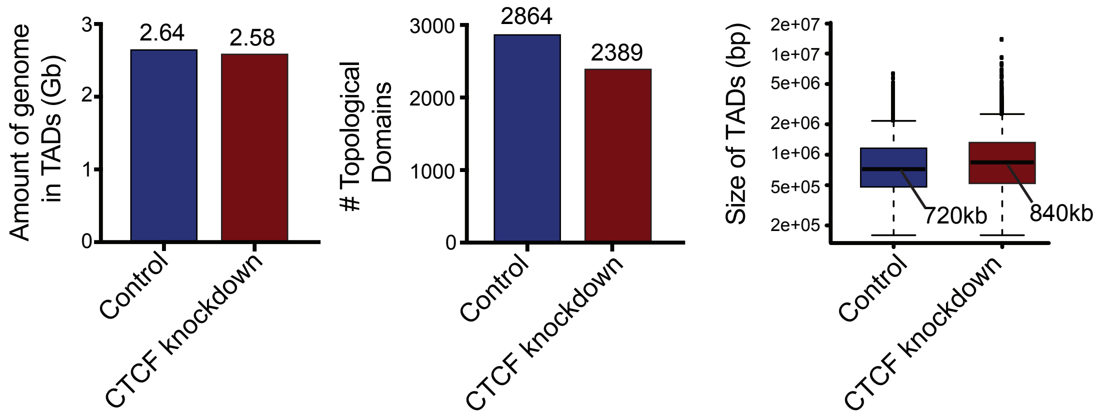


**Supplementary Figure 9: Comparison of TAD metrics in control and CTCF knockdown conditions**
Left: Amount of genome contained in TADs. Middle: Total number of TADs. Right: Median size of TADs (centre line). Lower and upper box borders denote the first and third quartile ranges of the data, respectively. Whiskers extend an additional 1.5 interquartile ranges.

# Supplementary Figure 10

**a**

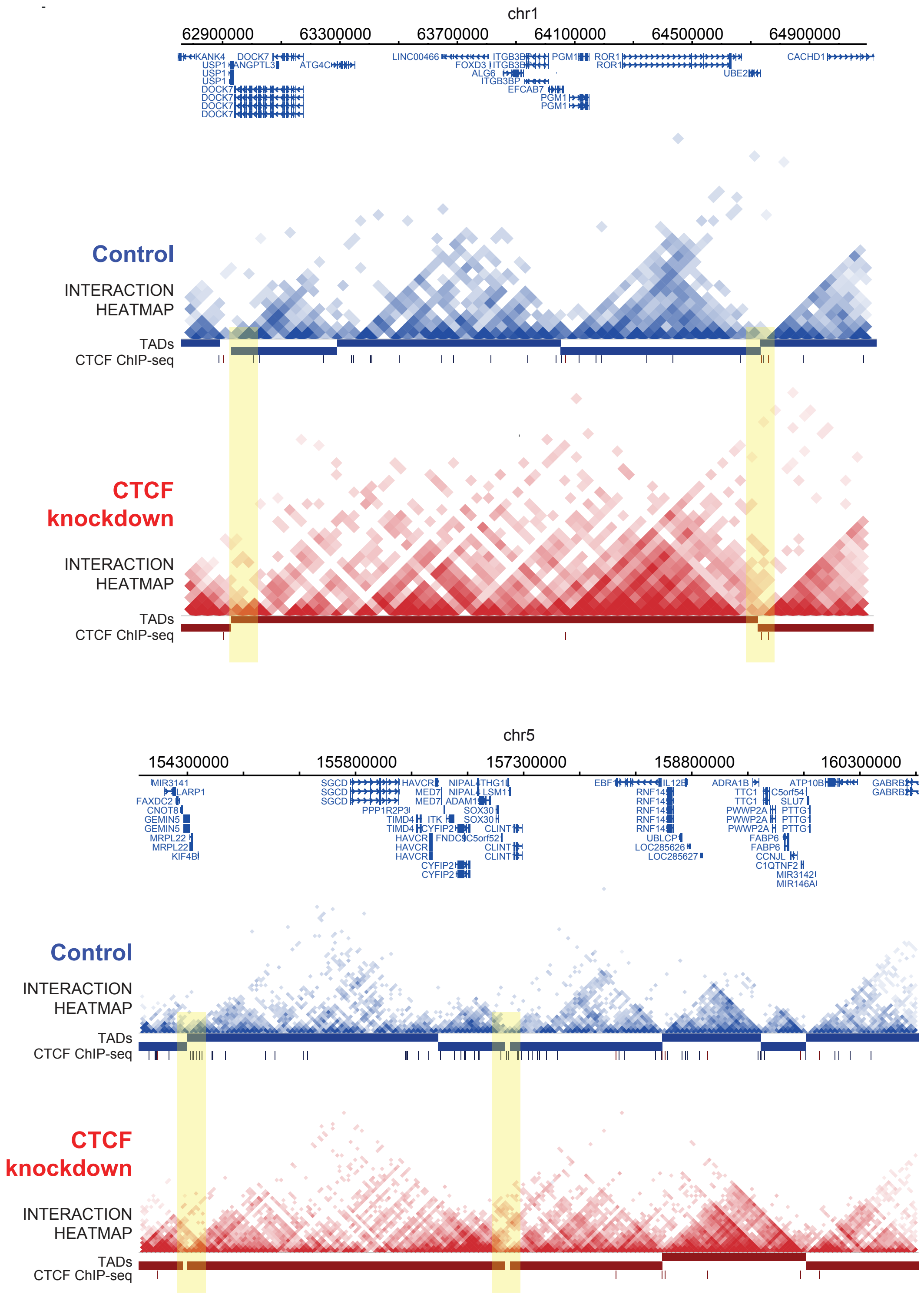

**b**

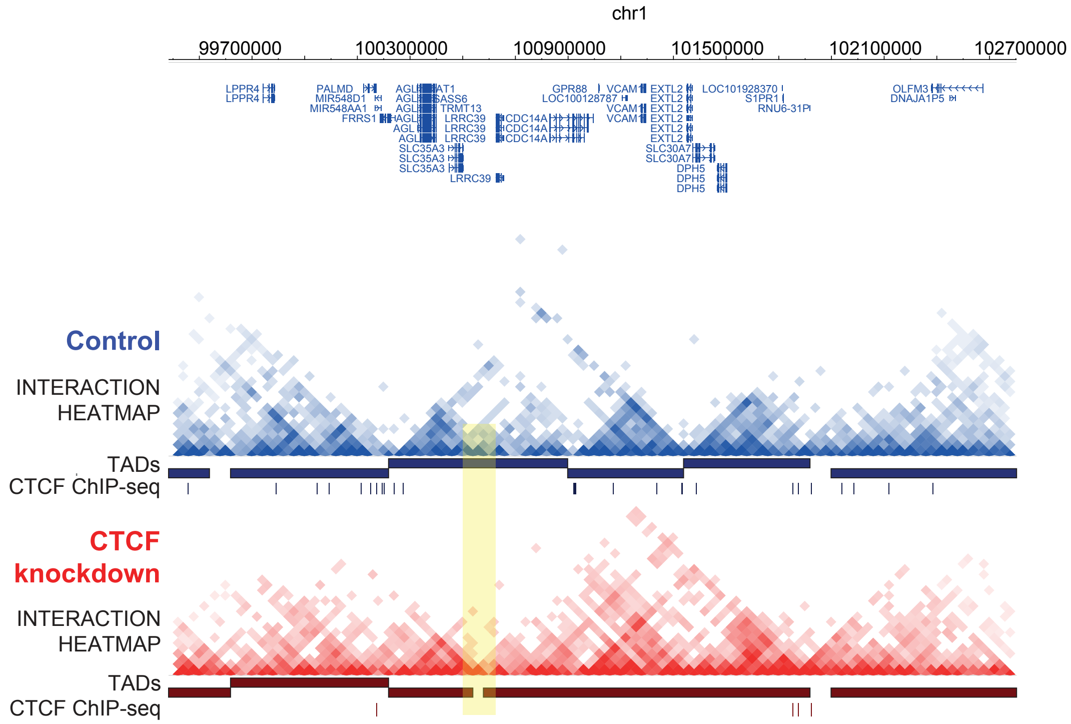


**Supplementary Figure 10: Depletion of CTCF leads to loss and gain of TAD boundaries examples**
**a**. Screenshots from Washu epigenome browser. Heatmaps, TADs and CTCF ChIP-seq data for 144hr control (blue) and CTCF (red) RNAi conditions in LNCaP cells demonstrating merging of TADs following CTCF knockdown and **b**. formation of new TAD boundaries following CTCF knockdown.

# Supplementary Figure 11

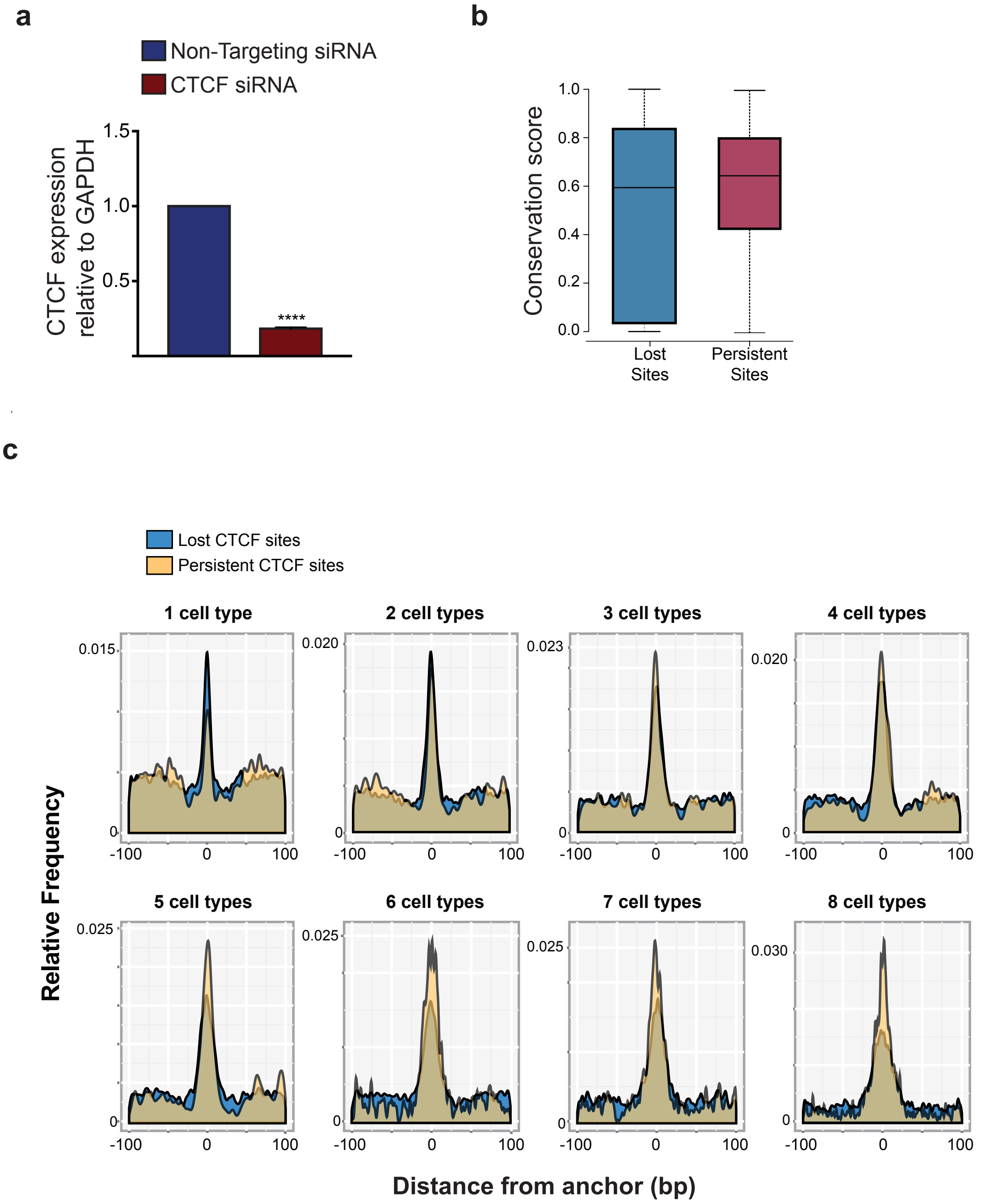


**Supplementary Figure 11: LNCaP persistent sites are present at cell-type constitutive chromatin conformation.**

**a**. qPCR measuring relative levels of CTCF mRNA for control siRNA and CTCF siRNA conditions at 144hrs following transfection with 20nM control and CTCF siRNA (two-tailed t-test, ****$p < 0.0001$). Error bars represent SE. Source data are provided as a Source Data file.

**b**. Bar blot of PhastCons conservation scores for lost and persistent CTCF sites. Median is indicated by the horizontal lines. Lower and upper box borders denote the first and third quartile ranges of the data, respectively. Whiskers extend an additional 1.5 interquartile ranges.

**c**. Loop anchor data for GM12878, K562, HeLa, IMR-90, HUVEC, NHEK, HMEC, KBM7 and HMEC cells was subset based on whether each loop anchor was cell-type specific (present in one cell line), common (present in two – seven cell lines) or cell-type constitutive (present in all eight cell lines). Positional enrichment of lost and stable CTCF sites was plotted for each subset. Lost sites are enriched at cell-type specific loop anchors. Stable sites are enriched at cell-type constitutive loop anchors.

**Supplementary Tables**

| Primer | Sequence |
|---|---|
| **KLK_CTCF1_ChIP_F** | GCAACCAATTTCTGGCTGAGA |
| **KLK_CTCF1_ChIP_R** | TTCTTGGCTGCAAAACTCCA |
| **KLK_CTCF2_ChIP_F** | ACACACCCCGCCATATGCT |
| **KLK_CTCF2_ChIP_R** | TCCATCTTTGGCGGGATTC |
| **KLK_CTCF3_ChIP_F** | CCTATGAGGCTGCCATCCC |
| **KLK_CTCF3_ChIP_R** | AGGACTTGGCAACCCTCCA |
| **KLK_CTCF4_ChIP_F** | AGGAGGCAGAAAAACCCACA |
| **KLK_CTCF4_ChIP_R** | CAATTGATGCCACCTATCGGT |
| **KLK_CTCF5_ChIP_F** | ACTGGTAAGGTTGGGTCAACGT |
| **KLK_CTCF5_ChIP_R** | CGGTCCAATGCCACCTAGA |
| **KLK_CTCF6_ChIP_F** | CAATTTCTTAGGATCTCTACCCGG |
| **KLK_CTCF6_ChIP_R** | GATGTGTCTGCAGCCTGCAT |
| **KLK_CTCF7_ChIP_F** | TGCGCCTACAACTGTGCAAT |
| **KLK_CTCF7_ChIP_R** | ACAACCTTTAGTCCTCCCCGA |
| **KLK_CTCF8_ChIP_F** | ATGCCCCTCCCACAACTTTC |
| **KLK_CTCF8_ChIP_R** | CATTTGCTCTATCGCGCCA |
| **KLK_CTCF9_ChIP_F** | CACCGGACCCTGGCTTCTA |
| **KLK_CTCF9_ChIP_R** | TCACTCCTCTCCCGCTGATT |
| **KLK_CTCF10_ChIP_F** | GGCTACCATATTGGCCAGCA |
| **KLK_CTCF10_ChIP_R** | TTATTGTTTTTCTCCAACTTCCATGA |
| **KLK_CTCF11_ChIP_F** | TGATGTCAATGACCACCGGA |
| **KLK_CTCF11_ChIP_R** | GTGCTCTTTTCGTCTCTGGCC |
| **KLK_CTCF12_ChIP_F** | GCACCCCCTACAGCTCATTC |
| **KLK_CTCF12_ChIP_R** | CAACAGTGGGTCCAATTTCCA |
| **KLK_CTCF13_ChIP_F** | CCATCTCCCCGGGAAGTTT |
| **KLK_CTCF13_ChIP_R** | CGGGTCTCAGGGTTGCTGT |
| **WNT2_F** | GGTGCAAGGAAATTACAGGGC |
| **WNT2_R** | TCCATCTGCCGACTTTCTGG |

**Supplementary Table 1:** Primers for CHIP-qPCR at *KLK* region and negative control

| Fragment ID | Primer sequence |
|---|---|
| **3CKLKFrag1FW** | AACCCAGGAGGTGAAAGAAAGTT |
| **3CKLKFrag2** | TTTGGAAATGGAATCTTGCTTTG |
| **3CKLKFrag2FW** | TGCACTCTAGCCTGGGCAATA |
| **3CKLKFrag3FW** | CAGATGTCCTGCGGACTCTTG |
| **3CKLKFrag4** | ACGTGGGCTCCAATATCCAA |
| **3CKLKFrag5** | GAAAGAATGTATAATCCTATCAGTGGAAA |
| **3CKLKFrag7** | GAATCTGTGGTTAGCCTGATCTTGA |
| **3CKLKFrag8** | CCAGATCCCCATATGTGAAACC |
| **3CKLKFrag9** | CTCTTACCAGGGTCTCCCAAAA |
| **3CKLKFrag13** | CCACGCAGCAGATGAGCAT |
| **3CKLKFrag14** | CACTCCCAACCCAGAATCCA |
| **3CKLKFrag18** | TTGTACAGCAGATAGCCTTGCAA |
| **3CKLKFrag23** | CAGCATTTACAGCTTACCTTCACGTA |
| **3CKLKFrag25** | CTCAGCCTCTGTGCCTTCTGT |
| **3CKLKFrag31** | TGCCGACCACCTTGATTTCT |
| **3CKLKFrag33** | GCAAATCAGTGCCCAAGAAAGT |
| **3CKLKFrag36** | AGGGCACCATGGCAAGATC |
| **3CKLKFrag39** | TTGAGATTCACAAGCAGGAGTCA |
| **3CKLKFrag40** | CGGGACTCCACCCCTTGT |
| **3CKLKFrag41** | TGTGGCTCTGCCCAGCAT |
| **3CKLKFrag42** | TGACCAGGCCCTCTCCAA |
| **3CKLKFrag46** | CGGTCAAAAGACTAAATTACAACACATT |
| **3CKLKFrag49** | GAGTCAGGGAGAGGGAGAGTGAA |
| **3CKLKFrag54** | CCATATAAAACCTAGACCCCTCCTAAAT |
| **3CKLKFrag54FW** | TCAAGGAGTGTCTGGTGTTTATACG |
| **3CKLKFrag56** | CAGGAGGCTGAGGCTTGAAC |
| **3CKLKFrag56FW** | CGAAGCCTTTCATTCTTTCCATA |
| **3CKLKFrag58** | GTCTCCACAGATTGCCAAAGG |
| **3CKLKFrag59** | AACAAAAGGCACCCTGTGCTT |
| **3CKLKBAIT** | GCATATCGGAAGTGCTCAGTAAAAA |

**Supplementary Table 2:** 3C Primers

| Site to be deleted | Input sequences for CRISPR Tool |
|---|---|
| **CTCF persistent site 1 (chr19: 51,425,541-51,425,856)** | CACAGAGAAGCTCCCGGGTTGTTTGCAGAAAACAGC GGGCAGGTGAGAGCGCTGGGCTGGGCACTCCCCAAG CTCGGAGCAAGTGCGCCTACAACTGTGCA**ATACTGC CACCTAGTGGAGC**CGGAGAGACATACTCGGGGAGG ACTAAAGGTTGTGGGCCCAGGGTTCTCTTGCGCCAGT CACCCTGGGCCGCAGCATCCGGCTCAGGATCCAGAA ACGGGAGCAGAGGTGGCAGGA |
| **CTCF persistent site 2 (chr19: 51,433,008-51,433,454)** | TGGGATTACCGGCGTGAGCCACCGCGCCCGGCCCTG GATTCCAAATTTCATGCCCCTCCCACAACTTTCCCAG GTCCCAGTTCAGTTCTTGCAA**AGGCCTCCAGGTGGC GCGAT**AGAGCAAATGGTGGATTCCGCTAACTTGGGT TTACAATTTCTTCCTGGGAGGCTTGCAGGGTGGGGCA CTTACTTGAACGTGCACACGAAACACCAGGGGATCT TATGGA |

**Supplementary Table 3:** Input DNA sequences for CRISPR Design Tool

| | GUIDE sequence |
|---|---|
| **chr19: 51,425,541-51,425,856_Guide1a_F** | **CACC**GTCTCTCCGGCTCCACTAGG |
| **chr19: 51,425,541-51,425,856_Guide1a_R** | **AAAC**CCTAGTGGAGCCGGAGAGAC |
| **chr19: 51,425,541-51,425,856_Guide1b_F** | **CACC**GTCGGGGAGGACTAAAGGTTG |
| **chr19: 51,425,541-51,425,856_Guide1b_R** | **AAAC**CAACCTTTAGTCCTCCCCGAC |
| **chr19: 51,433,008-51,433,454_Guide1a_F** | **CACC**GTGCTCTATCGCGCCACCTGG |
| **chr19: 51,433,008-51,433,454_Guide1a_R** | **AAAC**CCAGGTGGCGCGATAGAGCAC |
| **chr19: 51,433,008-51,433,454_Guide1b_F** | **CACC**GATGGTGGATTCCGCTAACTT |
| **chr19: 51,433,008-51,433,454_Guide1b_R** | **AAAC**AAGTTAGCGGAATCCACCATC |

**Supplementary Table 4:** gRNAs generated for CRISPR-Cas9n experiments

| Primer | Forward | Reverse |
|---|---|---|
| ***ALAS1*** | CCACACACCCCAGATGATGA | CCCACTTGCTTCCATGTGACT |
| ***CTCF*** | ATGCGCTCTAAGAAAGAAGATTCCT | CTCATCCTCATTGTCGTCCAGA |
| ***KLK15*** | TGGCTTCTCCTCACTCTCTCCTT | CCTTCCAGCAACTTGTCACCAT |
| ***KLK3*** | TGTGGGTCCCGGTTGTCTT | CCCAGCCTCCCACAATCC |
| ***KLK2*** | ACAGCTGCCCATTGCCTAAA | GTGTCTTCAGGCTCAAACAGGTT |
| ***KLKP1*** | CATCCTCACTGGGTGCTCACTAC | CTATGGTGCTGGCTAGTTGATCA |
| ***KLK4*** | GAGGGCACGACCAGAAGGA | AAGACACAAGGCCCTGCAAGT |
| ***KLK5*** | AGATGACACCATGTTCTGCGC | CACAGGCCCCCCAGAATC |
| ***KLK6*** | GGGATGAGAAGTACGGGAAGG | TCGGAGGTGGTCTCCACATAC |
| ***KLK7*** | CATCCCCGACTCCAAGAAAA | GCAGGGTACCTCTGCACACC |
| ***KLK8*** | AAGAGCAGGAACATCCACGTCT | GAATCAGTAGGTGACCCCGC |
| ***KLK9*** | AGTCACCCTGGCAGGAACCT | AGTCACCCTGGCAGGAACCT |
| ***KLK10*** | GAGTTGGGTGCTGACGGC | AAGAAGCAGCAGGTGGTCATC |
| ***KLK11*** | TCTGGTGCTCAATGATGGTGA | GGCAGCACGTCCAGC |
| ***KLK12*** | GCTGTGAGTTACGCCCACACT | CATCTTTTTGCTCCTGTGTGTTCTT |
| ***KLK13*** | GAGACGATGCCATACAGTGTTCTG | GAGACGATGCCATACAGTGTTCTG |
| ***KLK14*** | GATGTTCACGCATTGCAGAGA | GACCTCCTGCCGAGTGTCAG |
| ***CTU1*** | TAGGAAGTTCATGAGCACGGTCT | CCGCGTGGGAGCCAC |
| ***SIGLEC9*** | CCGCCTCAGAACTTGACCAT | GAGCCATTTCCCAAGACTGTG |

**Supplementary Table 5:** qPCR expression primers
