# Supplementary material for: Constitutively bound CTCF sites maintain 3D chromatin architecture and long-range epigenetically regulated domains
Source: Nat Commun. 2020 Jan 7;11:54. doi: 10.1038/s41467-019-13753-7 (PMC6946690; doi:10.1038/s41467-019-13753-7)
Supplement: Supplementary file 3 — Reporting Summary [file 41467_2019_13753_MOESM3_ESM.pdf]

nature research

Corresponding author(s): Susan J. Clark

Last updated by author(s): Nov 14, 2019

Reporting Summary

Nature Research wishes to improve the reproducibility of the work that we publish. This form provides structure for consistency and transparency in reporting. For further information on Nature Research policies, see Authors & Referees and the Editorial Policy Checklist.

Statistics

For all statistical analyses, confirm that the following items are present in the figure legend, table legend, main text, or Methods section.

n/a	Confirmed	
☐	☒	The exact sample size (n) for each experimental group/condition, given as a discrete number and unit of measurement
☐	☒	A statement on whether measurements were taken from distinct samples or whether the same sample was measured repeatedly
☐	☒	The statistical test(s) used AND whether they are one- or two-sided<br>*Only common tests should be described solely by name; describe more complex techniques in the Methods section.*
☒	☐	A description of all covariates tested
☐	☒	A description of any assumptions or corrections, such as tests of normality and adjustment for multiple comparisons
☐	☒	A full description of the statistical parameters including central tendency (e.g. means) or other basic estimates (e.g. regression coefficient) AND variation (e.g. standard deviation) or associated estimates of uncertainty (e.g. confidence intervals)
☐	☒	For null hypothesis testing, the test statistic (e.g. F, t, r) with confidence intervals, effect sizes, degrees of freedom and P value noted<br>*Give P values as exact values whenever suitable.*
☒	☐	For Bayesian analysis, information on the choice of priors and Markov chain Monte Carlo settings
☒	☐	For hierarchical and complex designs, identification of the appropriate level for tests and full reporting of outcomes
☒	☐	Estimates of effect sizes (e.g. Cohen's d, Pearson's r), indicating how they were calculated

Our web collection on statistics for biologists contains articles on many of the points above.

Software and code

Policy information about availability of computer code

Data collection

Affymetrix Array
- Bioconducter packages, "oligo" and "limma"

Hi-C
- NGSane v0.5.2
- HiCuP v0.5.2
- Fit-HiC ((Ay et al. 2014; Libbrecht et al. 2015)
- HiCorrector v1.1
- “domain-caller” (Bing Ren: http://chromosome.sdsc.edu/mouse/hi-c/download.html)

ChIP-seq
- NGSane v0.5.2.0
- Bowtie v1.1.0
- Macs2 v2.1.0
- Peakranger
- diffBind v1.14.4
- HOMER v4.10.3

Low Input ChIP-seq
-BWA v0.7.13
-picard-tools v2.3.0
-samtools v1.3
-java v1.8
-fastqc v0.11.5
-cutadapt v2.4
-Macs2 v2.1.2

October 2018

Data analysis

All analysis (except for low cell ChIP-seq) was performed using open source software. All software code used to analyze the data for this study is publicly available as described in the methods section.
R software and packages: R v3.2.5, ggplot2_1.0.1, EnsDb.Hsapiens.v75_0.99.12, GenomicFeatures_1.20.1, Rsamtools_1.20.4, BSgenome.Hsapiens.UCSC.hg19_1.4.0, rtracklayer_1.28.4, DiffBind_1.14.4, limma_3.24.10, Repitools_1.14.0, aaRon_0.9.5 (Github), BSgenome_1.36.0, GenomicRanges_1.20.5.

The software and policy for low cell ChIP-seq has been uploaded with the manuscript submission.

For manuscripts utilizing custom algorithms or software that are central to the research but not yet described in published literature, software must be made available to editors/reviewers. We strongly encourage code deposition in a community repository (e.g. GitHub). See the Nature Research guidelines for submitting code & software for further information.

Data

Policy information about availability of data

All manuscripts must include a data availability statement. This statement should provide the following information, where applicable:

- Accession codes, unique identifiers, or web links for publicly available datasets
- A list of figures that have associated raw data
- A description of any restrictions on data availability

The datasets generated and/or analysed during the current study have been uploaded to the Gene Expression Omnibus repository, GEO number GSE125641, Link https://www.ncbi.nlm.nih.gov/geo/query/acc.cgi?acc=GSE125641. Reviewer token: sxcdaiqgfrkrvmn

Field-specific reporting

Please select the one below that is the best fit for your research. If you are not sure, read the appropriate sections before making your selection.

☒ Life sciences ☐ Behavioural & social sciences ☐ Ecological, evolutionary & environmental sciences

For a reference copy of the document with all sections, see nature.com/documents/nr-reporting-summary-flat.pdf

Life sciences study design

All studies must disclose on these points even when the disclosure is negative.

Sample size	All experiments were performed in duplicate or triplicate to access statistical significance.
Data exclusions	No data was excluded from analysis.
Replication	All experiments were performed in duplicate or triplicate. All replicate experiments were concordant.
Randomization	No randomisation was required in our study as no human or animal subjects were used.
Blinding	No blinding was required in our study as no human or animal subjects were used.

Reporting for specific materials, systems and methods

We require information from authors about some types of materials, experimental systems and methods used in many studies. Here, indicate whether each material, system or method listed is relevant to your study. If you are not sure if a list item applies to your research, read the appropriate section before selecting a response.

Materials & experimental systems

n/a	Involved in the study	
☐	☒	Antibodies
☐	☒	Eukaryotic cell lines
☒	☐	Palaeontology
☒	☐	Animals and other organisms
☒	☐	Human research participants
☒	☐	Clinical data

Methods

n/a	Involved in the study	
☐	☒	ChIP-seq
☒	☐	Flow cytometry
☒	☐	MRI-based neuroimaging

Antibodies

Antibodies used	CTCF (#07-729, Millipore) Rad21 (#ab992, Abcam), H3K4me3 (#39159, Active Motif), H3K27ac (#39133, Active Motif).
Validation	H3K4me3 (Active Motif #39159) and H3K27ac (Active Motif, #39133) antibodies are validated for ChIP-seq in the Antibody Validation Database (Egelhofer et al. (2010)). CTCF antibody (Millipore #07-729) has been validated for ChIP-seq by Millipore (http://www.merckmillipore.com/AU/en/product/Anti-CTCF-Antibody,MM_NF-07-729) and has been used in numerous publications. Rad21 antibody (Abcam, #ab992) has been validated by (Abcam https://www.abcam.com/rad21-antibody-chip-grade-ab992.html) and has been used in numerous publications.

Eukaryotic cell lines

Policy information about cell lines

Cell line source(s)	LNCaP prostate cancer cells (ATCC #CRL-1740). IMR90 cells (ATCC #CCL-186)
Authentication	LNCaP cells were authenticated by short-tandem repeat profiling. LNCaP profiling was performed by CellBank Australia, Westmead, NSW, Australia. IMR90 cells were authenticated by ATCC.
Mycoplasma contamination	All cell lines used in-house tested negative for mycoplasma using the MycoAlert Mycoplasma Detection Kit (Lonza, #LT07-318).
Commonly misidentified lines (See ICLAC register)	No cell lines from the ICLAC register were used.

ChIP-seq

Data deposition

☒ Confirm that both raw and final processed data have been deposited in a public database such as GEO.

☒ Confirm that you have deposited or provided access to graph files (e.g. BED files) for the called peaks.

Data access links<br>*May remain private before publication.*	https://www.ncbi.nlm.nih.gov/geo/query/acc.cgi?acc=GSE125641.
Files in database submission	GSM3579298 LNCaP_Control siRNA 144hrs H3K4me3 Rep1<br>GSM3579299 LNCaP_Control siRNA 144hrs H3K4me3 Rep2<br>GSM3579300 LNCaP_Control siRNA 144hrs H3K27ac Rep1<br>GSM3579301 LNCaP_Control siRNA 144hrs H3K27ac Rep2<br>GSM3579302 LNCaP_Control siRNA 144hrs CTCF Rep1<br>GSM3579303 LNCaP_Control siRNA 144hrs CTCF Rep2<br>GSM3579304 LNCaP_CTCF siRNA 144hrs H3K4me3 Rep1<br>GSM3579305 LNCaP_CTCF siRNA 144hrs H3K4me3 Rep2<br>GSM3579306 LNCaP_CTCF siRNA 144hrs H3K27ac Rep1<br>GSM3579307 LNCaP_CTCF siRNA 144hrs H3K27ac Rep2<br>GSM3579308 LNCaP_CTCF siRNA 144hrs CTCF Rep1<br>GSM3579309 LNCaP_CTCF siRNA 144hrs CTCF Rep2<br>GSM3579310 IMR90_Control siRNA 144hrs CTCF Rep1<br>GSM3579311 IMR90_Control siRNA 144hrs CTCF Rep2<br>GSM3579312 IMR90_CTCF siRNA 144hrs CTCF Rep1<br>GSM3579313 IMR90_CTCF siRNA 144hrs CTCF Rep2<br>GSM4087661 Low Cell Control siRNA 144hrs CTCF Rep1<br>GSM4087662 Low Cell Control siRNA 144hrs CTCF Rep2<br>GSM4087663 Low Cell CTCF siRNA 144hrs CTCF Rep1<br>GSM4087664 Low Cell CTCF siRNA 144hrs CTCF Rep2
Genome browser session (e.g. UCSC)	Do not have saved session

Methodology

Replicates	Each ChIP was performed in duplicate and showed good agreement. For stringency, only peaks present in both replicates were used for analysis.
Sequencing depth	GSM3579298 LNCaP_Control siRNA 144hrs H3K4me3 Rep1- 24980316 - 50bp, SE<br>GSM3579299 LNCaP_Control siRNA 144hrs H3K4me3 Rep2- 31291689- 50bp, SE<br>GSM3579300 LNCaP_Control siRNA 144hrs H3K27ac Rep1 - 22495305- 50bp, SE<br>GSM3579301 LNCaP_Control siRNA 144hrs H3K27ac Rep2 - 18846544- 50bp, SE<br>GSM3579302 LNCaP_Control siRNA 144hrs CTCF Rep1 - 18954655- 50bp, SE,Sequenced a second time: 26729456 - 50bp, SE<br>GSM3579303 LNCaP_Control siRNA 144hrs CTCF Rep2 - 18905204- 50bp, SE,Sequenced a second time: 19868059 - 50bp, SE<br>GSM3579304 LNCaP_CTCF siRNA 144hrs H3K4me3 Rep1- 27083599- 50bp, SE

GSM3579305 LNCaP_CTCF siRNA 144hrs H3K4me3 Rep2- 24765159- 50bp, SE
GSM3579306 LNCaP_CTCF siRNA 144hrs H3K27ac Rep1- 25405794- 50bp, SE
GSM3579307 LNCaP_CTCF siRNA 144hrs H3K27ac Rep2 - 15980934- 50bp, SE
GSM3579308 LNCaP_CTCF siRNA 144hrs CTCF Rep1 - 5551699- 50bp, SE, Sequenced a second time: 6726158 - 50bp, SE
GSM3579309 LNCaP_CTCF siRNA 144hrs CTCF Rep2 - 17611276- 50bp, SE, Sequenced a second time: 24365613 - 50bp, SE
GSM3579310 IMR90_Control siRNA 144hrs CTCF Rep1 41037440- 50bp, SE
GSM3579311 IMR90_Control siRNA 144hrs CTCF Rep2- 32842941- 50bp, SE
GSM3579312 IMR90_CTCF siRNA 144hrs CTCF Rep1- 48095718- 50bp, SE
GSM3579313 IMR90_CTCF siRNA 144hrs CTCF Rep2 - 44749302- 50bp, SE
GSM4087661 Low Cell Control siRNA 144hrs CTCF Rep1 - 14856529-75bp, SE
GSM4087662 Low Cell Control siRNA 144hrs CTCF Rep2 - 16276110-75bp, SE
GSM4087663 Low Cell CTCF siRNA 144hrs CTCF Rep1 - 17923283-75bp, SE
GSM4087664 Low Cell CTCF siRNA 144hrs CTCF Rep2 - 14185084 - 75bp, SE

Antibodies

CTCF (#07-729, Millipore), H3K4me3 (#39159, Active Motif), H3K27ac (#39133, Active Motif).

Peak calling parameters

CTCF peaks were called using MACS2 with FDR 0.05 assuming a fragment size of 200bp. Histone mod peaks were called using peak ranger software (Feng et al, 2011) using default parameters.

Data quality

ChIP-seq libraries were validated by qPCR (n=3). All peaks are below the Peak Ranger and MACS2 FDR cut off.

Software

Software ChIP-seq reads were aligned to hg19 using bowtie v1.1.0 allowing up to 3 mismatches, discarding ambiguous and clonal reads.
